# Evaluation of Microstructure and Mechanical Properties of Al-TiC Metal Matrix Composite Prepared by Conventional, Microwave and Spark Plasma Sintering Methods

**DOI:** 10.3390/ma10111255

**Published:** 2017-10-31

**Authors:** Ehsan Ghasali, Ali Fazili, Masoud Alizadeh, Kamyar Shirvanimoghaddam, Touradj Ebadzadeh

**Affiliations:** 1 Ceramic Department, Materials and Energy Research Center, Alborz, P.O. Box 31787-316 Karaj, Iran; afazili@merc.ac.ir (A.F.); malizadeh@merc.ac.ir (M.A.); t.ebadzadeh@merc.ac.ir (T.E.); 2 Carbon Nexus, Institute for Frontier Materials, Deakin University, Geelong, VIC 3216, Australia

**Keywords:** aluminum, titanium carbide, microwave, spark plasma sintering

## Abstract

In this research, the mechanical properties and microstructure of Al-15 wt % TiC composite samples prepared by spark plasma, microwave, and conventional sintering were investigated. The sintering process was performed by the speak plasma sintering (SPS) technique, microwave and conventional furnaces at 400 °C, 600 °C, and 700 °C, respectively. The results showed that sintered samples by SPS have the highest relative density (99% of theoretical density), bending strength (291 ± 12 MPa), and hardness (253 ± 23 HV). The X-ray diffraction (XRD) investigations showed the formation of TiO_2_ from the surface layer decomposition of TiC particles. Scanning electron microscopy (SEM) micrographs demonstrated uniform distribution of reinforcement particles in all sintered samples. The SEM/EDS analysis revealed the formation of TiO_2_ around the porous TiC particles.

## 1. Introduction

Aluminum matrix composites (AMCs) have a distinct place in automotive and aerospace industries due to their superior properties such as high specific strength and Young’s modulus, low cost, and good wear resistance [1,2,3,4]. Extensive studies have been done on AMCs to improve their mechanical and tribological properties because of their potential in many applications. So far, different ceramic materials such as Al_2_O_3_ [5], SiC [6], MgO [7], WC [8], B_4_C [9], ZrB2 [10], SiC-TiC [11], ZrSiO_4_ [12], TiB_2_ [13], and TiO_2_ [14] have been used as reinforcing agents within an aluminum matrix.

TiC is a carbide with high hardness, high elastic modulus, high thermal conductivity, and low density, which make it a proper reinforcement for aluminum composites [15,16]. There are contradictory observations about the wettability and reactivity of TiC with molten aluminum [17,18,19,20,21,22,23]. According to research studies, it seems that TiC shows reasonable wettability and controllable reactivity. Hence, wettability and reactivity are affective qualities on reinforcement/matrix adhesion, and, therefore, the final properties of the composite are highly influenced by these parameters due to the load transfer from the matrix to reinforcements [24,25].

Many techniques have been used to fabricate AMCs such as casting routes [26], powder metallurgy [27], in situ synthesis [28], and spray forming [29]. Each fabrication method has its own advantages and disadvantages. For example, casting methods offer a low cost process as a unique advantage; on the other hand, the non-uniform dispersion of the reinforcements and unwanted interfacial reactions represent the main problems [30,31,32,33,34,35,36].

The processing temperature in the powder metallurgy (PM) method is low compared casting methods, so they are promising methods to achieve uniform dispersion of the reinforcement particles. Sintering steps in PM process such as blending and compacting have a brilliance effect on the final properties of product [37,38,39]. SPS, microwave sintering, laser sintering, and hot isostatic pressing are advanced sintering techniques in comparison with other conventional methods. Each of these methods has specific effects on the final properties of products. SPS introduces better properties in comparison with microwave and conventional sintering due to the specific advantages of this method such as short sintering time, application of pressure during sintering process, formation of spark between particles, vacuum sintering, etc. [40,41,42,43,44,45].

M. Ali et al. [46] used the conventional method for Al-TiC nano composite sintering at 500, 550, and 600 °C in a protective argon atmosphere, and reported a reasonable hardness.

A. Kennedy et al. [47] investigated the effect of different fabrication methods (powder metallurgy-hot isostatic press (HIP), flux-assisted casting method, extruding mixed powders) on the interfacial bonding and mechanical properties of Al-TiC composite.

According to the best of the author’s knowledge, no reports have yet been made on the effect of various types of heat sources on the sintering and properties of Al-TiC composites. The purpose of the present work is to compare the influence of different sintering routes on the microstructure and mechanical properties of Al-TiC metal matrix composites.

## 2. Results and Discussion

**Figure 1:**
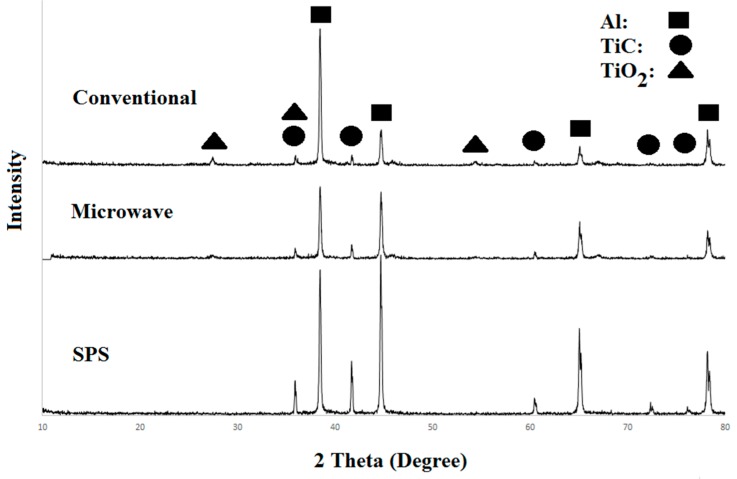
XRD (X-ray diffraction) patterns of SPS (speak plasma sintering), microwave, and conventional samples sintered at 400 °C, 600 °C, and 700 °C.

Figure 1 shows the XRD patterns of conventional, microwave, and SPS sintered samples. In the precision of XRD, the only crystalline phases detected after sintering by SPS were aluminum and titanium carbide. In both microwave and conventional sintered samples, besides aluminum and titanium carbide, TiO_2_ was also identified.

S. Shimada et al. [48] reported the oxidation behavior of TiC in dry and wet oxygen at 300–400 °C. The results showed the decomposition of TiC and the formation of TiO_2_ according to the following reaction [49,50]:

TiC + 2O_2_ = TiO_2_ + CO_2_ (1)

On the other hand, J. Viala [51] investigated the stability of TiC in molten aluminum and proposed the following reaction:

7Al + TiC = Al_3_Ti + Al_4_C_3_ (2)

Viala reported two possible explanations for the oxidation of TiC and the formation of TiO_2_ in the presence of aluminum. The first one is the sintering of samples takes place in the solid state phase according to the reaction number (2) (if this reaction takes place). The second one is the formation of an ultrathin oxide layer (Al_2_O_3_) around aluminum particles that prevents Al from reaching TiC particles. It is worth mentioning that in the conventional or microwave sintered samples, the formation of small amounts of Al_4_C_3_ is possible but is not detected by XRD. There are at least two possible sources for the existence of oxygen in the conventional and microwave processes. One is the oxygen trapped between Al and TiC particles during uniaxial pressing of samples and the second is the diffusion of oxygen through the graphite bed. In samples sintered by SPS, the oxidation of TiC was not observed (Figure 1) which can be attributed to the low sintering temperature and the use of the vacuum during operation.

**Figure 2:**
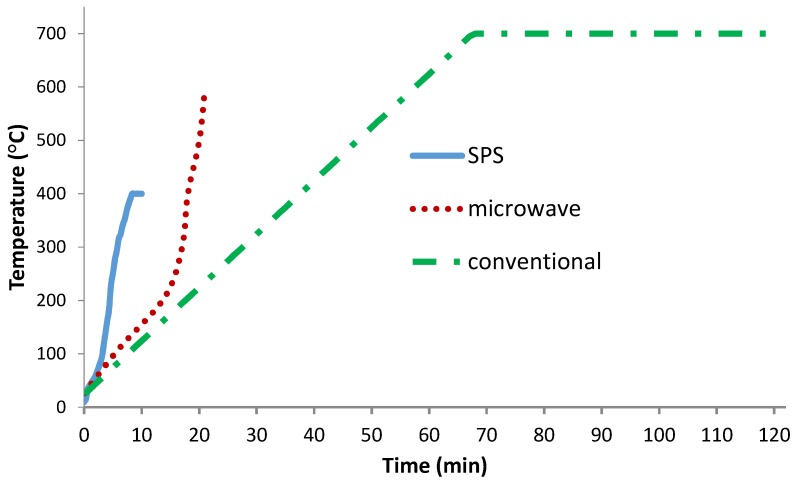
Temperature-time curve for conventional, microwave, and spark plasma sintering processes.

Figure 2 shows the changes of temperature versus time for samples sintered in conventional and microwave furnaces and by SPS.

As can be seen in Figure 2, the total sintering time for SPS, microwave, and conventional heating is almost 10, 21, and 120 min, respectively. The sintering time and temperature can directly have an effect on the final properties of specimens such as the formation of new compounds, crystallite size, and grains size. Subsequently, each of these parameters can cause changes in the microstructure and mechanical properties of sintered specimens. It seems that a fast heating rate with a low sintering temperature as well as the vacuum condition in SPS, compared with conventional and microwave heating, is the main reason for the existence of Al and TiC particles without the TiO_2_ layer.

**Figure 3:**
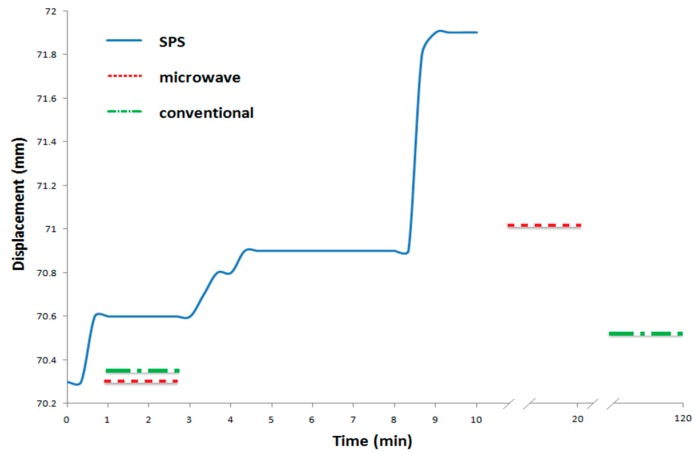
Displacement changes vs sintering time for SPS sintering.

**Figure 4:**
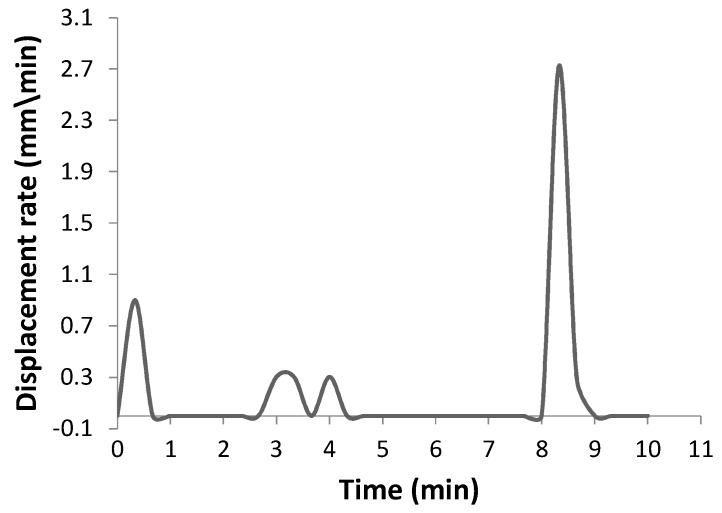
Displacement rate vs sintering time for the SPS sample.

The shrinkage profile of microwave and conventional sintered samples were transformed to displacement changes of SPS specimens due to the lack of monitoring of the sintering. So, the initial size of microwave and conventional specimens was compared with the initial punch position of the SPS apparatus (Figure 3). The punch displacement of the SPS sample in Figure 3 indicates three important changes. The first area between 0 and 1 min of sintering time shows the rearrangement of particles and the creation of sparks between them. The second area between 3 and 4.5 min implies that the sintering stage begins. There are no significant changes in punch displacement between 4.5 and 8 min, indicating that the density is not increased further. The third area between 8 and 9 min demonstrates increasing pressure from 10 to 40 MPa, indicating the maximum punch displacement, implying the final stage of sintering and resulting in the highest relative density. The abovementioned results show that SPS sintered samples had higher shrinkage compared to conventional and microwave sintered samples. Figure 4 shows the displacement rate changes versus sintering time, and there are three important areas that indicate sintering stages. The maximum shrinkage occurs at sintering time between 8 and 9 min, while the applied pressure increases from 10 to 40 MPa. No detectable changes were seen in the shrinkage after a sintering time of 9 min, which means that the maximum shrinkage had taken place at this time (Figure 3 and Figure 4).

The use of these diagrams could help us to better understand the sintering behavior and shrinkage of specimens. Besides these two, other parameters such as the quality of the interface between matrix particles, the uniform distribution of reinforcement particles in the matrix, interfacial reaction products, and grain size have important roles in the final properties of prepared samples.

**Figure 5:**
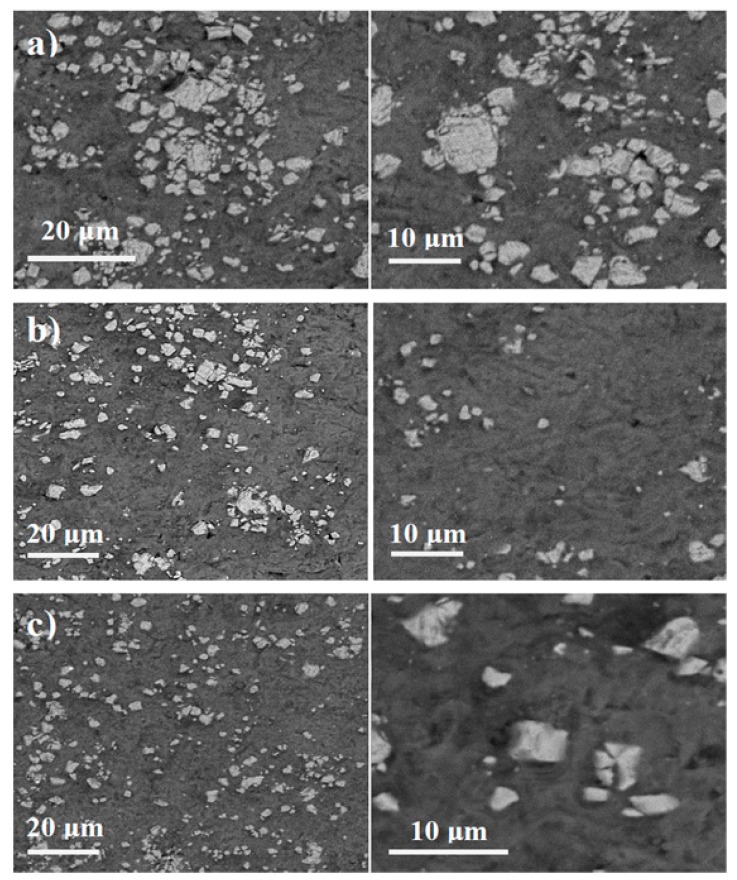
SEM micrograph of: (**a**) conventional; (**b**) microwave; and (**c**) SPS sintered samples.

Figure 5 shows the distribution of reinforcing particles in SPS, microwave, and conventional sintered samples. As observed, more microcracks are found around TiC particles in the microstructure of conventional and microwave sintered samples rather than SPS sintered samples. As discussed earlier in the cause of observed TiO_2_ peaks in XRD patterns, one possible reason for the presence of these micro-cracks is the formation of CO_2_ as a result of the surface decomposition of TiC by oxygen trapped in the small spaces between the particles and/or oxygen diffusion into the sample from the furnace atmosphere. Other possible reasons can be related to the existence of an initial thin protective Al_2_O_3_ layer that covers the aluminum particles.

**Figure 6:**
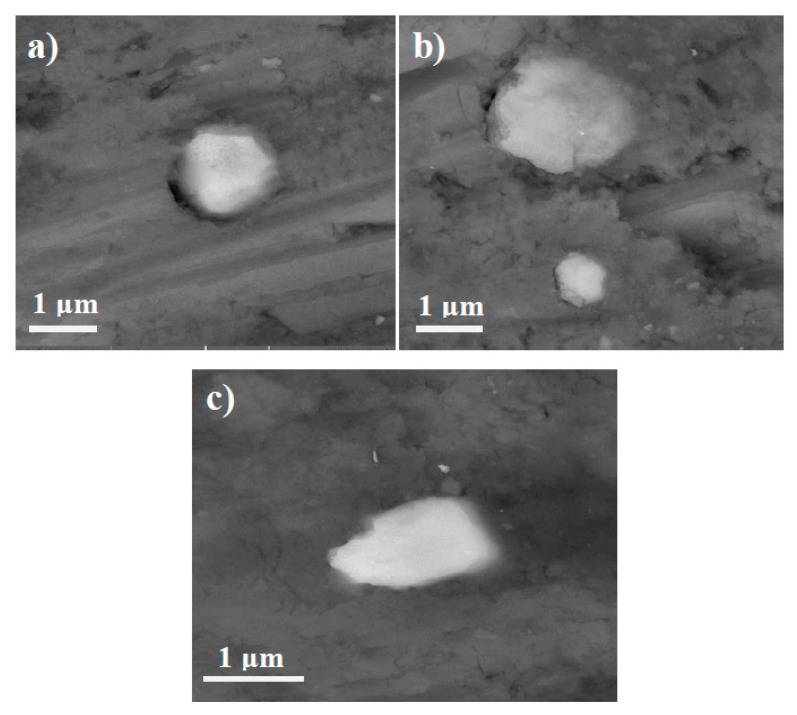
FESEM micrographs of: (**a**) conventional; (**b**) microwave; and (**c**) SPS sintered samples.

Field emission scanning electron microscopy (FESEM) images of samples sintered at different sintering routes (Figure 6) take a closer look at the separation of TiC particles from the matrix in microwave and conventional sintered samples, but not for TiC particles in the sample sintered by SPS (Figure 6c).

It seems that, not only the decomposition of TiC particles in conventional and microwave processes leads to particle-matrix separation, but the higher sintering temperature in conventional and microwave methods could also encourage this separation due to high mismatch in the thermal expansion coefficient of Al and TiC particles.

**Figure 7:**
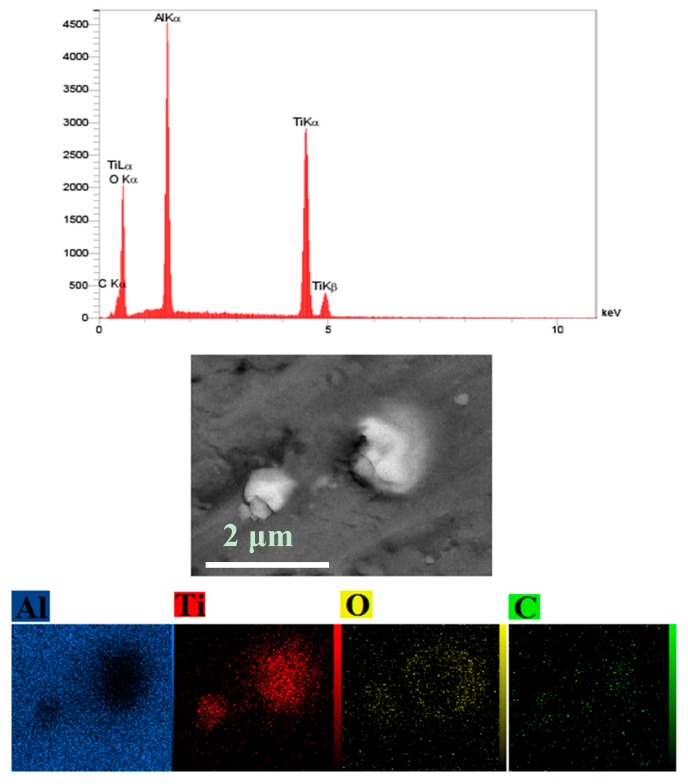
Backscattered and EDS elemental mapping of the microwave sintered sample.

**Figure 8:**
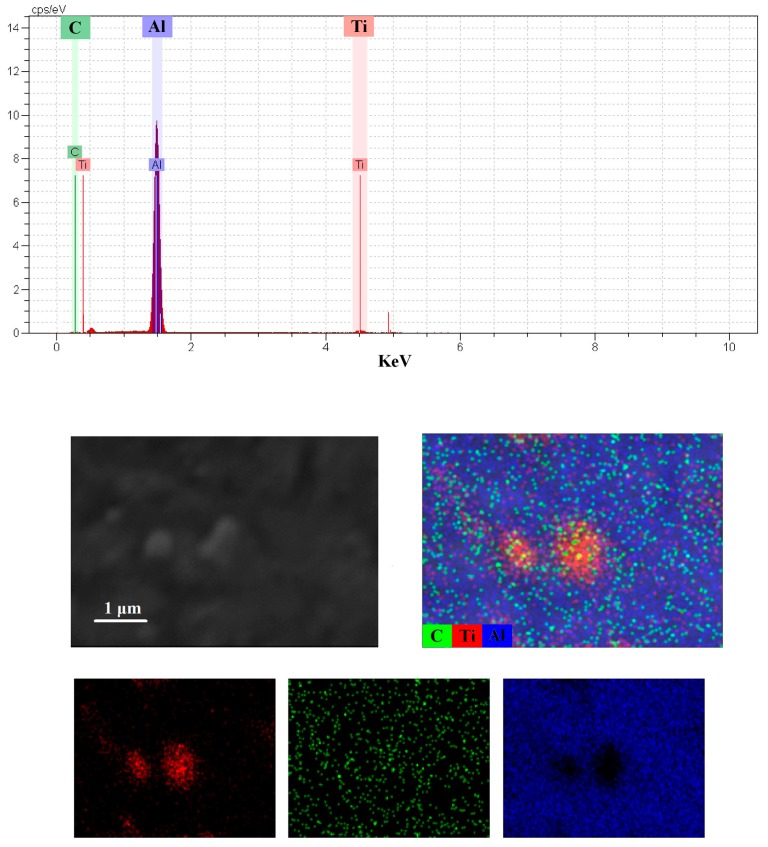
Backscattered and EDS elemental mapping of the spark plasma sintered sample.

Figure 7 and Figure 8 show FESEM micrographs and EDS elemental mapping of microwave and spark plasma sintered samples. A comparative look at these figures demonstrates the existence of elemental oxygen around TiC particles in the microwave process (Figure 7), while no oxygen was found in the microstructure of SPS sintered samples. The formation of an interfacial layer of TiO_2_ around TiC particles was confirmed by investigation of the EDS elemental maps. It is worth mentioning that the formation of more TiO_2_ in the conventional process is possible due to the higher sintering time and temperature of conventional rather than microwave sintered samples (Figure 1).

Table 1 presents the physical and mechanical properties of SPS, microwave, and conventional sintered samples including density, bending strength, and microhardness. Since the TiC decomposes, the bulk density rather than the relative density was measured.

As can be seen from Table 1, the maximum density and mechanical properties were obtained for samples sintered by SPS. With regard to the theoretical density of the Al-15 wt % TiC composite (2.895 gr/cm^3^), if no decomposition of SPS sample occurs, then the relative density of the sintered sample can be calculated as 99.4%, which is acceptable as a nearly full dense specimen. Comparison of density and other mechanical properties of samples sintered in microwave and conventional furnaces with SPS show that the decomposition of TiC in samples sintered in microwave and conventional furnaces intensively affects the bending strength and microhardness of the samples. The lowest amount of bending strength and microhardness was obtained for conventionally sintered samples, most probably due to a higher sintering temperature and more decomposition of reinforcement particles that result in more defects in the microstructure as well as weak mechanical properties. The mechanical properties of microwave sintered samples were lower and higher than those of SPS and conventional sintered samples, respectively.

**Figure 9:**
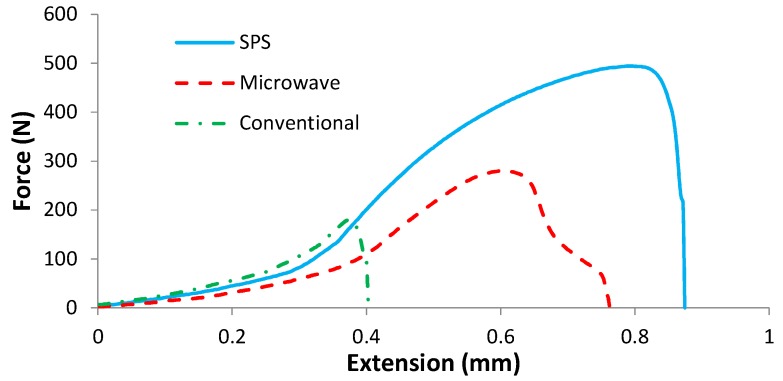
Load-extension curve for SPS, microwave, and conventional sintered samples.

Figure 9 emphasizes the load-extension behavior of samples during bending strength examination and shows a sharp increase in toughness for the SPS sintered sample, confirmed by the mechanical properties values in Table 1.

## 3. Experimental Procedures

### 3.1. Preparation of Composite

Aluminum (Fluka, particle size of 100–200 μm, 99% purity, St. Louis, MO, USA) and TiC (Fluka-89480, average particle size of 2 μm, 99.5% purity) powders were used as starting materials. Then, 15 wt % TiC was mixed with aluminum in ethanol media using a high-energy mill, and then the mixture was dried at 70 °C. The dried powders were inserted into a bar shape (5 mm × 5 mm × 25 mm) and then uniaxially pressed at 250 MPa and sintered in conventional and microwave furnaces. The mixed powders were directly used in the SPS process without pre-forming.

### 3.2. Sintering Process

Conventional sintering was carried out at 700 °C with heating rate of 10 °C/min in a graphite bed for 1 h. An assembled microwave furnace (900 W and 2.45 GHz) was used for the sintering of composites. The sintering temperature of 600 °C was detected by an optical pyrometer (Raytek, Model: RAYR312MSCL2G, Wilmington, NC, USA). The microwave processing was conducted with SiC susceptors using a graphite bed. Microwave sintering was performed with an average heating rate of 30 °C/min in a thin layer of graphite powders to prevent the oxidation of the aluminum composite. The mixed powders were directly inserted in the graphite die with a diameter of 30 mm in the SPS (SPS-20T-10, EasyFashion Industry, Changsha, China) case and the sintering process was conducted at 400 °C with initial and final pressures of 10 (during increasing temperature) and 40 MPa (at a maximum temperature of 400 °C), respectively. The pulse cycle in this work was 12 ms ON, 2 ms OFF, and a high DC current (0–1200 A) applied to the sample.

### 3.3. Microstructure and Mechanical Properties

Phases identification was carried out by XRD (Philips X’ Pert System, Philips, Amsterdam, The Netherlands) using a Cu kα (λ = 1.5404 A°) source and an image plate detector over the 2θ range from 10° to 80° in reflection geometry. The bulk density of sintered samples was measured using the Archimedes’ Principle. The three-point bending flexural test was used to examine the strength of sintered samples. The bending strength samples fabricated by SPS were cut from a disc with a diameter of 30 mm. Vickers microhardness values of the sintered samples were calculated using at least 10 successive indentations for each sample by a MKV-h21 Microhardness Tester under a load of 10 N for 15 s. Microstructural characterization of sintered samples was examined using SEM (SEM VEGA//TESCAN-XMU, Czech Republic and S360 Cambridge, Cambridge, UK) and FESEM (MIRA 3 TESCAN, Czech Republic and S360 Cambridge, Cambridge, UK), both equipped with an energy dispersive spectrometer (EDS).

Details of the material preparation procedure and the sintering process as well as microstructural characterization studies are the same as those reported previously [11].

## 4. Conclusions

Al-TiC metal matrix composites were fabricated successfully via spark plasma, microwave, and conventional sintering methods. The SPS method with utilizing pressure and a low sintering temperature led to proper microstructure and mechanical properties, in comparison to the other sintering methods. Microwave and conventional sintering, due to the higher sintering temperature and time, resulted in the decomposition of TiC and the formation of TiO_2_ and CO_2_ that exhausted CO_2_ gas-produced porosities in the microstructure of the samples. The SPS sintered samples achieved almost full density with the highest mechanical properties at a lower sintering temperature and time than was required for the microwave and conventional sintered samples.

## Figures and Tables

**Table 1:** Physical and mechanical properties of SPS, microwave, and conventional sintered samples.

Sample	Density (gr/cm^3^)	Bending Strength (MPa)	Microhardness (Vickers)
SPS	2.88 ± 0.01	291 ± 12	253 ± 23
Microwave	2.76 ± 0.03	134 ± 21	110 ± 15
Conventional	2.60 ± 0.02	95 ± 9	80 ± 13
